# A balanced perspective on unbalanced growth and thymineless death

**DOI:** 10.3389/fmicb.2015.00504

**Published:** 2015-06-05

**Authors:** Philip C. Hanawalt

**Affiliations:** Department of Biology, Stanford University, Stanford, CA, USA

**Keywords:** DNA replication cycle, DNA degradation, replication origin, DNA repair, thymine starvation, transcription, genomic uracil, RecQ

## Abstract

The early history of the esoteric phenomenon of thymineless death (TLD) is recounted, from the pioneering discovery by Seymour Cohen and Hazel Barner, through my graduate studies at Yale and postdoctoral research in Copenhagen. My principal contribution was the discovery that restricted synthesis of protein and RNA permits cultures of *Escherichia coli* to complete their DNA replication cycles without initiating new ones, and that cells held in this physiological state are immune to the lethality of thymine deprivation; unbalanced growth is not the fundamental cause of TLD. The successful synchronization of the DNA replication cycle contributed to formulation of the replicon concept. Studies at Stanford revealed a specific requirement for transcription and led to the discovery of a TLD-resistant mutant in a new gene, termed *recQ*, with important homologs in humans and most other organisms. The lessons learned from research on TLD underscore the value of basic research in bacterial systems that can have profound implications for human health.

## Introduction

This review constitutes a reflection upon my early experiences in biomedical research, with particular focus upon the enigmatic phenomenon of thymineless death (TLD) that began during my graduate study and that still commands my intrigue. I came to appreciate how advances in science and their promotion are coupled to the personalities and aspirations of the investigators. While a discovery may incorporate novel insights, it always builds upon the contributions from predecessors and usually includes elements of serendipity. I will candidly relate the collaborative interactions among some pioneering investigators who have strived to achieve a mechanistic understanding of TLD, while the ultimate comprehension still eludes their grasp.

Upon graduation from Oberlin College with a major in physics, I joined the biophysics program at Yale for graduate study in 1954. That was an exciting era; the Watson–Crick DNA structure and a plausible model for DNA replication had just emerged, but we did not have a clue about the functions of RNA or its relationships to DNA. My first research mentor, Harold Morowitz, suggested that I should synchronize the division cycle in cultures of the bacterium, *Escherichia coli* (*E. coli*), so that we could study the time-course of biochemical events from one division to the next. Unfortunately, a published synchronization procedure involving glucose starvation was not reproducible by us or by anyone else. Although I retained my interest in the cell cycle, I then joined the laboratory of Richard Setlow, with whom I had just completed an outstanding spectroscopy course. I wanted to explore the application of short-wavelength ultraviolet light (UV) to probe macromolecular activities in bacterial cells through action spectra. I developed an approach to resolve the cellular components of DNA, RNA, and phospholipids so that I could follow their respective syntheses by labeling with ^32^P orthophosphate (Hanawalt, 1959). However, I also wanted to follow DNA synthesis at higher specificity with ^14^C-labeled thymine, so I needed to improve the efficiency of its exogenous incorporation. In a literature search I found a timely paper describing a thymine-requiring bacterium (Barner and Cohen, 1954).

Seymour Cohen was characterizing *E. coli* 15T–, later shown to lack functional thymidylate synthetase. This auxotrophic mutant, originally isolated by Raymond Roepke from UV-irradiated *E. coli* strain 15, had been acquired from Joseph Gots at the University of Pennsylvania. Unlike the biostatic consequences of starvation for most required growth factors, such as uracil or cytosine, *E. coli* 15T– suffered a striking loss of viability when thymine was omitted from the defined growth medium, which may explain the scarcity of this type of mutant. The lethality was initially attributed to “unbalanced growth,” since the bacteria continued to grow in mass while cell division was curtailed and hardly any DNA synthesis was evident (Cohen and Barner, 1954); however, the cells in minimal medium survived if the sole carbon source, glucose, was omitted, indicating that metabolism was required for TLD. The suggestion that the inhibition of DNA synthesis by UV might also kill cells by unbalanced growth sparked my interest. Balanced growth, in contrast to unbalanced growth, was defined as the physiological condition over a time interval during which every extensive property of the growing system increased by the same factor (Campbell, 1957).

## TLD Studies at Yale

Cohen generously provided *E. coli* 15T–, and my research followed upon his work, with an eventual thesis entitled: “Macromolecular synthesis in *Escherichia coli* during conditions of unbalanced growth,” in which I compared the effects of thymine starvation to those caused by short-wavelength UV irradiation (Hanawalt, 1958). I confirmed that during thymine starvation the bacteria stopped dividing and formed long filaments, similar to those observed following UV irradiation (Deering and Setlow, 1957). The optical density (turbidity) continued to follow that of cultures in balanced growth for one division period or a little longer, while viability remained constant for about 30 min before it decreased exponentially at roughly 90% per division period. RNA synthesis in the absence of thymine was linear for two division periods before declining, while protein synthesis (followed by labeling with ^14^C-proline or ^35^SO_4_) leveled off earlier. Unlike the case for thymine starvation, DNA synthesis eventually recovered following low doses of UV (Hanawalt and Setlow, 1960) and the lag in recovery could be shortened by exposing the cells to photoreactivating light (Hanawalt and Buehler, 1960). Of course, the ^32^P labeling protocol was needed to confirm that there was very little DNA synthesis in the thymine-starved cells (Hanawalt, 1959). Cohen and Barner had also established that the thymine analog, 5-bromouracil (5BU), could partially fulfill the thymine requirement and prolong the viability of *E. coli* 15T– (Cohen and Barner, 1956). The incorporation of deoxyuridine or 5BU during DNA synthesis *in vitro* was documented by Bessman et al. (1958). At that point in history we were unaware of any effects of thymine starvation on DNA structure or function and we did not yet know the nature of the DNA damage produced by UV.

## TLD Studies in Denmark

**FIGURE 1:**
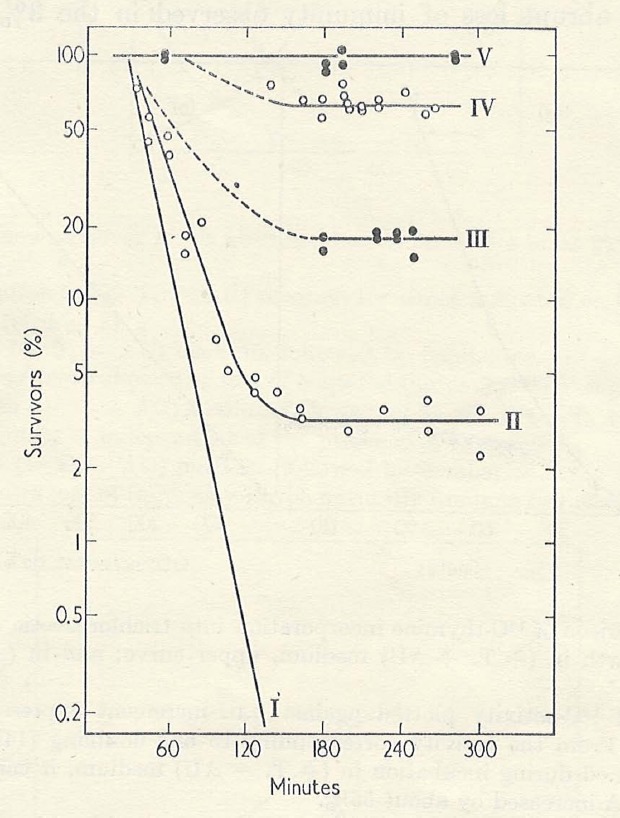
**Effect of pre-incubation with T but without AU on survival of *E. coli* TAU during subsequent incubation, –T–AU.** II; –T–AU (no pre-incubation): III; 30 min pre-incubation +T–AU: IV; 60 min pre-incubation +T–AU: V; 90 min pre-incubation +T–AU: I; Comparison curve for –T+AU, from another experiment. From Maaløe and Hanawalt (1961).

Upon completion of my Ph.D., I wanted to return to my earlier interest in synchronous growth, so I obtained an NIH postdoctoral fellowship to join Ole Maaløe in Denmark, where Gordon Lark, Elio Schaechter, and others had collaborated in studies of bacterial growth and division patterns upon changing media or shifting temperature. Maaløe was considered the international expert in bacterial synchronous growth, and he had just been appointed Professor/Chair of a newly established Department of Microbiology at the University of Copenhagen. Shortly before leaving Yale I received from Cohen a triple-mutant derivative of *E. coli* 15T– called “TAU,” auxotrophic for arginine (A) and uracil (U), as well as for thymine (T; Barner and Cohen, 1958). Maaløe offered no immediate suggestions for a research project upon my arrival in his lab, so I simply continued with the TLD studies I had begun at Yale. Although I recorded some interesting effects of incubation temperature on TLD, the most exciting results were those obtained when A and U were withheld (henceforth, “AU,” since both requirements were due to the same mutation in carbamoyl phosphate synthase). The first thing I noted was that although mass (turbidity) did not increase in the absence of AU, nearly all of the cells (∼97%) still suffered TLD. Thus, unbalanced growth at the level of cell mass in relation to DNA content could not be responsible for the lethality. In the presence of T but absence of AU, DNA synthesis continued for over an hour to achieve roughly a 40% increase before it leveled off. Interestingly, the fraction of the population resistant to TLD in the absence of AU also increased during that period of allowed DNA synthesis. By the time DNA synthesis had ceased, the entire population of cells had become immune to TLD, unless protein and RNA syntheses were again permitted (Figure 1). These results prompted me to suggest that the cellular DNA replication cycle could be completed in the absence of protein and RNA synthesis, but that protein and/or RNA synthesis were required for re-initiation of the cycle; and furthermore, that cells are only susceptible to TLD if they are actively replicating DNA. (This made logical sense: why would they need thymine if they were not replicating DNA? Of course, we did not yet know about *repair replication*!) I had inadvertently discovered a simple approach for synchronizing the DNA replication cycle in bacteria. At this point Maaløe became intensely interested in my project and I enjoyed more frequent discussions with him. We decided that we would need to use autoradiography to learn the status of DNA replication in individual cells in order to verify the model. Elio Schaechter had previously employed single cell autoradiography in Maaløe’s group to demonstrate that *E. coli* normally replicates DNA throughout most of the cell cycle (Schaechter et al., 1959). Unfortunately, the technology had not been retained in Maaløe’s lab when Schaechter departed. However, my graduate colleague from Yale, Bob Van Tubergen, was an expert in single cell autoradiography and had just joined Roy Markham’s group in Cambridge (Van Tubergen, 1961). That afforded an opportunity for me to visit England, to learn the autoradiographic procedures from Bob, and to carry out several preliminary experiments under his guidance. I then completed the single cell autoradiographic studies to confirm our model that individual cells terminated DNA synthesis at different times during incubation with T in the absence of AU. A new postdoc, Donald Cummings, soon joined Maaløe’s group and contributed experience in density-labeling DNA with 5BU to follow DNA synthesis. I was pleased to have Don as a collaborator because I appreciated the unique advantages of density-labeling for analysis of DNA replication (Meselson and Stahl, 1958). Density-labeling nascent DNA could tell us whether the DNA that had replicated in the absence of AU was able to begin a new round of synthesis before AU was again supplied. The studies of TLD in Copenhagen provided early insights into control of the normal DNA replication cycle in bacteria and contributed to the formulation of the concept of the replicon (Jacob et al., 1963).

Other researchers were also attracted to the TLD field, while Barner and Cohen (1956) continued their leadership with many additional contributions, including evidence for at least one synchronous division when thymine was re-added 30 min after starvation, the report that a single amino acid deficiency does not completely block TLD although it inhibits growth (Barner and Cohen, 1957), and the finding that deprivation of uracil (in a double-mutant for T and U) prolongs the lag period for TLD and induces RNA turnover (Barner and Cohen, 1958). The UV irradiation of cells prior to thymine starvation was shown to shorten the lag period before the onset of TLD, suggesting some similarities in the “cellular units” affected by the respective treatments (Gallant and Suskind, 1961). However, there was still no information on the nature of DNA damage due to either UV or thymine starvation.

## TLD Studies at Caltech

**FIGURE 2:**
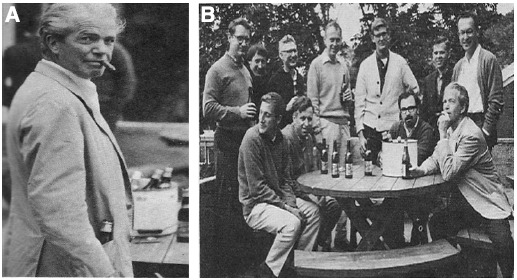
**Photos from Cold Spring Harbor Symposium Vol. XXXIII on “Replication of DNA in Micro-organisms” (1968). (A)** Professor Ole Maaløe, with his ever-present cigar. **(B)** Reunion of alumni from Ole Maaløe’s research group Standing: P. Hanawalt, J. Clark, C. Levinthal, J. Watson, P. Kuempel, C. Helmstetter, D. Glaser Seated: K. G. Lark, M. Schaechter, S. Cooper, O. Maaløe, with cigar.

With an interest in learning more about biophysical approaches for characterizing nucleic acids, and hoping to discover what thymine starvation actually did to DNA, I joined the group of Robert Sinsheimer at the California Institute of Technology for a third-year postdoc. Shortly after my arrival at Caltech in late 1960 I heard about an upcoming Cold Spring Harbor Symposium on “Cellular Regulatory Mechanisms,” for which Maaløe was an invited speaker. Unfortunately, the conference had been oversubscribed so it was too late for me to attend. I learned later that my mentor had featured our TLD project in his lecture and in the published proceedings as sole author, and had acknowledged his research assistant, Jens Ole Rostock, for the autoradiography (Maaløe, 1961). The results were simultaneously published in two journal articles (Hanawalt et al., 1961; Maaløe and Hanawalt, 1961). The authorship of the second paper included not only Don Cummings for the 5BU studies but also Elio Schaechter, who had not participated in the project and whom I had not even met. (That was my first encounter with “courtesy authorship.”) Although these were disappointing experiences, I learned important lessons from them about appropriate attribution of contributions in the dissemination of scientific advances. Seven years later, at the 1968 Cold Spring Harbor Symposium on “Replication of DNA in Microorganisms,” two of my graduate students gave invited talks on their own work, and I included four additional graduate students and two postdocs as co-authors for my presentation (Hanawalt et al., 1968). I also convened a reunion of alumni from Maaløe’s group for beer and informal discussions during the conference (Figure 2).

Although I learned a lot at Caltech from productive interactions with Sinsheimer, Max Delbrück, Matt Meselson, Jerry Vinograd, and their students, my search for DNA alterations during TLD did not reveal anything significant. The most exciting new information that year was the report that UV irradiation caused thymine to form covalent dimers (Beukers and Berends, 1960), and these were soon shown to be the predominant photoproducts in cellular DNA; so there was finally a specific lesion to study in relation to DNA synthesis inhibition and damage processing in UV-irradiated bacteria.

## TLD Studies at Stanford

I joined the Biophysics Laboratory at Stanford University in September 1961 with a primary focus on DNA replication in UV-irradiated *E. coli*, but also with ongoing TLD studies. I then acquired a new multiple auxotroph of *E. coli* 15T– from Richard Wax that required methionine, tryptophan, proline, and arginine as well as thymine, and had a more stringent uracil requirement than that in strain TAU. (I named it TAU-bar, simply to avoid confusion with TAU). The removal of uracil greatly reduced TLD and implicated mRNA transcription in the lethality; however, I emphasized that one must distinguish between the event that causes killing and an event that converts the cell from the immune state to the susceptible state (Hanawalt, 1963). Gallant and Suskind (1962), in studies of *E. coli B3,* had concluded that TLD is correlated with unbalanced synthesis of RNA. In later studies we showed that the transcription inhibitor, rifampicin, essentially eliminates TLD, while also preventing the DNA strand breaks we had observed during thymine deprivation (Nakayama and Hanawalt, 1975). The accumulation of strand breaks had been seen, or not, by a number of other researchers using different bacterial strains and different conditions, but it was difficult to assess whether this could be a cause or an effect of TLD, in part because some of the strains carried inducible prophage or colicin factors (Freifelder, 1969; Ahmad et al., 1998 for review). Following upon our discovery of repair replication in UV-irradiated *E. coli* (Pettijohn and Hanawalt, 1964), we documented the non-conservative repair mode of DNA replication in thymine-starved bacteria (Pauling and Hanawalt, 1965), and we speculated that transcription was somehow responsible for introducing strand breaks or gaps that required thymine for their repair. An obvious hypothesis was that, in the absence of thymidine, DNA polymerases incorporated deoxyuridine and this would lead to a futile cycle of removal and resynthesis, as the uracil in DNA is recognized by glycosylases (e.g., Ung) for repair. The pool of deoxyuridylate was reported to increase markedly during thymine starvation (Goulian and Beck, 1966). Meanwhile, adding to the complexity of models, it was shown that initiation of replication from the chromosomal origin occurred before completion of the previous replication cycle during thymine starvation, and that amino acid starvation prevented what was called “*premature* initiation” (Pritchard and Lark, 1964). Cells growing in very low thymine concentrations exhibited increased *thymineless mutagenesis* and required an extended period for completion of chromosomal DNA synthesis (Zaritsky and Pritchard, 1971). Since thymine limitation can be seen as the *approach* to thymine exhaustion, some features of thymine deficiency may already be apparent, as exemplified by the more recent (40 years later!) results of Zaritsky et al. (2011), in which delayed initiations during thymine limitation are ascribed to possible “collapsing replisomes” and double strand breaks in the origin region.

Donachie and Hobbs (1967) had proposed that the lethality during TLD could result from sensitivity to plating on nutrient agar, but Nakayama and Couch (1973) established that the lethal event must occur *during the period of thymine starvation*, as they assayed viability entirely in the liquid phase, through application of the Poisson distribution to dilute suspensions of the bacteria.

Thymineless death was demonstrated in a member of the simplest class of living cells, *Mycoplasma laidlawii B* (Smith and Hanawalt, 1968) and in the most UV-resistant of known bacteria, *Deinococcus radiodurans*, which suffered TLD with roughly the same kinetics as *E. coli*, thereby challenging the hypothesis that the DNA damage due to thymine deprivation was similar to that due to UV (Little and Hanawalt, 1973). TLD was also documented in the eukaryote, *Saccharomyces cerevisiae* (Barclay and Little, 1978; Little, 1985), and of course, eventually in cultured mammalian cells. Goulian et al. (1985) studied mammalian cells treated with methotrexate, which inhibits dihydrofolate reductase to result in thymidine deficiency, and found a greater than 1000-fold increase in dUTP (normally <0.3 nM) associated with the drop in intracellular dTTP. They documented the incorporation of uracil and fragmentation of newly replicated DNA together with lethality, supporting the hypothesis that an elevated concentration of dUTP in mammalian cells resulted in TLD through a mechanism of uracil incorporation and removal during DNA repair (Goulian et al., 1985). For current reviews on uracil in DNA and its processing, see Krokan et al. (2014)and Williams and Kunkel (2014).

An undergraduate from Cornell University, Jack Nunberg, spent the summer of 1971 in our laboratory to study the DNA degradation associated with thymine deprivation, following the report of selective degradation of nascent DNA during TLD in *Bacillis subtilis* (Reiter and Ramareddy, 1970). Breitman et al. (1972) had also documented loss of thymine from DNA and an early increase in deoxyribose during thymine starvation in *E. coli* TAU-bar. We had previously documented preferential loss of nascent DNA in UV-irradiated *E. coli* (Hanawalt and Brempelis, 1967), and many years later we learned that the RecQ helicase and RecJ 5–3 exonuclease were responsible for that specific degradation of the lagging DNA strand (Courcelle and Hanawalt, 1999). Nunberg found that the *E. coli* CR34/74 temperature-sensitive DNA synthesis mutant (deficient in the DnaB helicase that operates at the replication fork), exhibited nascent DNA degradation at the restrictive temperature but without loss of viability. However, TLD at the restrictive temperature in CR34/74 did not differ significantly from that at the normal growth temperature; thus, active DNA replication forks are not required for TLD! Using *E. coli* TAU-bar prelabeled with ^14^C-thymine and pulse labeled with ^3^H-thymine, Nunberg also found that “bulk” DNA degradation but not nascent DNA degradation was eliminated by rifampicin, in parallel with the protection from TLD (Nunberg, 1971). We had previously documented turnover of deoxyribonucleotides in DNA at ∼0.02% per generation period at the restrictive temperature in *E. coli* CR34/74, and this was markedly suppressed by rifampicin, in support of our hypothesis that transcription may sometimes result in repairable single-strand breaks (Grivell et al., 1975).

Hiroaki Nakayama selected survivors from a thymine-starved *E. coli K12* strain to isolate a mutant that was remarkably resistant to TLD. Upon return to Kyushu University from Stanford, he named the gene *recQ* (for *Kyushu*!) and identified the product as a helicase (Nakayama et al., 1984). RecQ participates in the RecF recombination pathway, and it turns out that mutants in other genes in this pathway are also resistant to TLD, whereas *recBC* mutants are unusually sensitive (Nakayama et al., 1982, 1988). RecQ is the prototype for the large family of RecQ helicases found in many bacterial strains and most eukaryotes, and its discovery is one of many examples that validate the importance of basic research on seemingly esoteric topics for future advances in biomedical science. Human cells have 5 *RecQ* homologs; mutation in one causes premature aging, mutation in another causes high levels of sister-chromatid exchanges and mutation in a third also has a cancer prone phenotype. For reviews of RecQ, see Nakayama (2005) and Larsen and Hickson (2013).

Structural changes in DNA from thymine-starved *E. coli* were revealed by pulsed field gel electrophoresis following cell lysis in agarose gels and post-lysis treatment with restriction endonucleases. Non-migrating DNA fragments accumulated in cells during thymine deprivation. Interestingly, the amounts of specific migrating DNA fragments were reduced inversely in relation to their map distances from the replication origin (*oriC*). Electron microscopy of the non-migrating DNA revealed convoluted structures, with branching and single-strand tails. The accumulation of non-migrating DNA was largely dependent upon RecA and *recF*-family genes, including *recQ*, implying links between the non-migrating DNA and TLD (Nakayama et al., 1994). However, the question remained as to whether these observations were revealing causes or consequences of TLD.

In the early 1990s the TLD field went into “eclipse” for a decade, with sparse publication of bacterial work, although the number of clinically relevant papers began to increase (for examples, see Canman et al., 1992; Houghton et al., 1997; Ladner, 2001; Liao et al., 2005). The *stalemate* in mechanistic comprehension is highlighted by the fact that a drafted review on TLD by Shamim Ahmad, Raymond Devoret, Hiroaki Nakayama, and myself in 1988, was eventually completed and published by Ahmad and several other co-authors 10 years later (Ahmad et al., 1998).

## Re-Awakened Interest in TLD

Periodically, I would suggest to a student that there were many interesting things yet to be learned from the study of TLD, but very few took the “bait” until an undergraduate, Pam Morganroth, joined my group in her junior year to study genetic control of TLD, for graduation with Honors (Morganroth and Hanawalt, 2006). TLD was characterized in *E. coli* mutants deficient in DNA synthesis (*dnaA, dnaB, dnaC,* and *dnaE*) and in cells treated with hydroxyurea (HU) to inhibit replication. Substantial resistance to TLD was observed when replication initiation was inhibited (*dnaA* and *dnaC*), but little resistance was noted when only replication elongation was inhibited (*dnaE* and HU). Morganroth’s results confirmed those of Bouvier and Sicard (1975), who had reported TLD resistance in several other temperature sensitive *dnaA* and *dnaC* mutants at the non-permissive temperature. It was concluded that active replication elongation is not required for the major TLD pathway, consistent with the findings of Nunberg (1971). A *uvrA* mutant, deficient in both global and transcription coupled nucleotide excision repair, exhibited normal TLD, ruling out participation of that ubiquitous pathway. In addition, the *lexA3* mutation, causing deficient activation of the SOS genomic stress response, did not affect TLD. However, the later studies of Fonville et al. (2010) indicated that part of the sensitivity to thymine starvation is dependent upon the SOS response, through activation of *sulA* to block cell division, in contrast to Morganroth’s results. Further study resolved the discrepancy by establishing different genotypes in the respective strains employed and by showing inexplicably that TLD is inhibited by CsrA, a *c*arbon *s*torage *r*egulator, in cells lacking the SOS response (Hamilton et al., 2013). Reflecting upon the roles of the *recF*-family genes, it is provocative to consider the paradoxical observation that while RecF proteins operate at arrested replication forks in UV-irradiated cells to enhance survival, their *absence* enhances survival in thymine-starved cells (Courcelle, 2005).

A major breakthrough in our understanding of TLD resulted from the application of genome wide DNA microarrays by Sangurdekar et al. (2010), who revealed a striking loss of genetic material, spreading outward from the origin (*oriC*), that correlated with lethality during thymine starvation. TLD resistant mutants in the RecFORJQ pathway had correspondingly less damage in the origin region. Using the same approach, the loss of origin-specific DNA during TLD has been confirmed by Kuong and Kuzminov (2012). Fonville et al. (2010) employed FISH analyses to record loss of DNA near the origin during thymine deprivation as well as eventual loss near the replication terminus at late times. Martín and Guzmán (2011) have confirmed the initiation of new replication initiations at the origin during thymine deprivation that correlate with TLD, and that are inhibited by rifampicin. Chapters from the groups of Guzmân and Khodursky follow in this special collection of articles on The Bacterial Cell (Guzmân and Martîn, 2015; Ostrer et al., 2015). It is exciting as we appear to be closing in on the prime culprit for TLD at the replication origin. The ultimate answer to the mechanism of TLD will surely implicate the essential role of RNA polymerase in initiating the DNA replication cycle (Lark, 1972; Martîn et al., 2014) as well as the genomic instability due to incorporation of uracil into DNA during the initiation of DNA synthesis at *oriC*. TLD underlies the mechanisms of action of a number of clinically important drugs such as methotrexate, trimethoprim, and fluorouracil. It is more than likely that the insights derived from the continuing studies of TLD in bacteria will have translational impact for chemotherapies that depend upon TLD-like responses.

### Conflict of Interest Statement

The author declares that the research was conducted in the absence of any commercial or financial relationships that could be construed as a potential conflict of interest.
